# Predicting the effect of antidepressant treatment on relief from anxiety symptoms

**DOI:** 10.1192/j.eurpsy.2022.492

**Published:** 2022-09-01

**Authors:** A. Spinrad, D. Taliaz, R. Zoller

**Affiliations:** Taliaz LTD, Data Science, Tel Aviv, Israel

**Keywords:** Precision Medicine, Anxiety disorders, machine learning, Treatment Selection

## Abstract

**Introduction:**

Depression and anxiety disorders are among the most prevalent forms of mental illness, with antidepressants frequently used to treat them. Unfortunately, prescription of antidepressant medication is often inexact and relies on a long trial-and-error process.

**Objectives:**

Using machine Learning (ML) algorithms on readily obtainable clinical and demographic data of individuals diagnosed with depression with anxiety symptoms, we hypothesized that we will be able to derive models which will enable a more accurate treatment selection, focusing on relief from anxiety symptoms.

**Methods:**

Patients’ data from the Sequenced Treatment Alternatives to Relieve Depression (START*D) were filtered to include only those who have considerable anxiety symptoms. We then analyzed these patients’ response patterns, focusing on their anxious symptomology. Then, feature selection algorithms were applied to select the most predictive features for anxiety relief. Finally, we trained three ML models for three antidepressants: citalopram, sertraline and venlafaxine, using a training set of participants, and validated them on naïve validation and test datasets. These ML models were then compiled to create a predictive algorithm.

**Results:**

Validating the algorithm on the validation and test sets, our algorithm achieved a balanced accuracy of 64.8% (*p*<0.001), 79.2% (*p*<0.001) and 78.03% (*p*<0.001) for citalopram, sertraline and venlafaxine, respectively.

**Conclusions:**

Our findings support applying ML to accumulating data to achieve an improvement in the treatment of mood disorders. The algorithm we developed may be used as a tool to aid in the choice of antidepressant medication, specifically for depressed patients who exhibit prominent anxiety symptoms.

**Disclosure:**

Dekel Taliaz is the founder and CEO of Taliaz and reports stock ownership in Taliaz. Amit Spinrad and Roni Zoller serve as data scientists in Taliaz.

